# A systematic review of PTSD to the experience of psychosis: prevalence and associated factors

**DOI:** 10.1186/s12888-020-02999-x

**Published:** 2021-01-07

**Authors:** Georgina Buswell, Zoë Haime, Brynmor Lloyd-Evans, Jo Billings

**Affiliations:** University College London, London, England

**Keywords:** Psychosis, Trauma, PTSD, Prevalence

## Abstract

**Background:**

Psychosis can be a sufficiently traumatic event to lead to post-traumatic stress disorder (PTSD). Previous research has focussed on the trauma of first episode psychosis (FEP) and the only review to date of PTSD beyond the first episode period was not systematic and is potentially outdated.

**Methods:**

We searched electronic databases and reference lists using predetermined inclusion criteria to retrieve studies that reported prevalence rates and associated factors of psychosis-related PTSD across all stages of the course of psychosis. Studies were included if they measured PTSD specifically related to the experience of psychosis. Risk of bias was assessed using an adapted version of the Newcastle Ottawa Scale. Results were synthesised narratively.

**Results:**

Six papers met inclusion criteria. Prevalence estimates of psychosis-related PTSD varied from 14 to 47%. Studies either assessed first-episode samples or did not specify the number of episodes experienced. Depression was consistently associated with psychosis-related PTSD. Other potential associations included treatment-related factors, psychosis severity, childhood trauma, and individual psychosocial reactions to trauma.

**Conclusions:**

Psychosis-related PTSD is a common problem in people with psychosis. There is a lack of published research on this beyond first episode psychosis. Further research is needed on larger, more generalizable samples. Our results tentatively suggest that prevalence rates of psychosis-related PTSD have not reduced over the past decade despite ambitions to provide trauma-informed care.

Prospero registration number: CRD42019138750.

## Background

Psychosis affects approximately 3% of the general population [1] with the two most common symptoms being hallucinations (perceptions occurring in the absence of external stimuli, such as hearing voices that other people cannot hear) and delusions (fixed, false beliefs) [2]. The experience of psychotic symptoms such as distressing hallucinations or persecutory delusions, and associated treatment responses, including involuntary hospitalisation, restraint and forced medication, can be sufficiently traumatic to lead to the development of post-traumatic stress disorder (PTSD) [3, 4].

Psychosis-related PTSD can be difficult to detect. Many symptoms of psychosis and PTSD share similarities [5] and people with psychosis are often not assessed for trauma disorders [6]. Undiagnosed PTSD prevents access to appropriate treatment, impeding recovery from psychosis or leading to additional co-morbidities such as anxiety and depression, substance abuse and suicidality [7, 8]. There may be reciprocal effects between psychosis and PTSD due to an overlap in symptoms; untreated PTSD can potentially exacerbate positive symptoms of psychosis. Research has suggested that those with comorbid PTSD and psychosis have more severe positive symptoms, independent of other factors [9].

The first episode of psychosis has been described as particularly traumatic due to its novelty [10]. However, PTSD from psychosis might be more prevalent amongst those who have had multiple episodes, if they were sensitised by their earlier episodes (in line with trauma sensitization theory [11],). Those who have experienced multiple episodes of psychosis, and therefore potentially more traumatic experiences, might be at increased risk of developing PTSD compared to those who have experienced one episode.

Research into risk factors for PTSD generally have found PTSD to be most prevalent amongst those who have previous trauma histories, who experienced intense emotional reactions and dissociation during the trauma, and who lacked social support afterwards [12, 13]. To date, little research has explored whether risk factors are similar when the traumatic event is psychosis.

So far, research into psychosis-related PTSD has been synthesised in two reviews [14, 15]. Berry et al. (2013) [14] reviewed 24 studies published up until 2011 and reported prevalence rates of psychosis-related PTSD varying from 11 to 67%. Some evidence suggested the following factors were associated with the development of psychosis-related PTSD: trauma history, psychosis severity, affective symptoms (e.g. depression), particular treatment experiences and psychological variables such as appraisals and coping style. However, evidence for these factors was generally weak and inconsistent between studies.

In their implications for future research, Berry et al. (2013) [14] highlighted that studies using sensitive measures of trauma were required, to support the investigation of associations between past traumas and psychosis-related trauma. They argued that it was important for future research to separate out symptom-related and treatment-related PTSD as these are conceptually different. They also recognised that psychological processes, such as contextual integration, should be investigated as these form part of models of PTSD. Regarding clinical implications of their review, Berry et al. (2013) [14] suggested that rates of psychosis-related PTSD may be reduced by improving hospitalisation experiences: noting the much lower rates of psychosis-related PTSD in an inpatient unit that adopted a person-centred, therapeutic model. However, the results of this review need to be interpreted with caution as their review methodology was not systematic, therefore they may have missed important research which presented alternative perspectives.

Following the Berry et al. (2013) [14] review there has been an increasing interest in the provision of trauma-informed care (TIC) across mental health settings [16]. TIC involves the recognition of trauma histories and the impact these have on patients, the prevention of potentially traumatic care practices, the provision of care environments that feel safe, both physically and psychologically, and clinicians working collaboratively with patients, empowering and respecting them. Patients should also have access to trauma treatment where appropriate [17]. Well delivered TIC may in future lead to reductions of rates of PTSD (particularly those related to treatment experiences) which should be reflected in research studies, although could conversely lead to an increase in reported rates in clinical practice due to improvements in case identification. Staff training programmes in providing TIC are still being developed and evaluated [18, 19] and it is likely that evaluations of their efficacy will be complex to measure. Reductions in funding of mental health services over the past decade could present challenges to the advancement of TIC and it is not clear if TIC is being delivered in services. However, due to the increasing recognition of trauma in both research and clinical settings it is possible there has been an acceleration in trauma research since Berry et al. (2013) [14] conducted their final search in 2011. An update of the review by Berry et al. (2013) [14] is therefore needed.

The more recent review by Rodrigues and Anderson (2017) [15] systematically searched for papers that reported prevalence rates and associated factors in first-episode psychosis (FEP) samples only. Of their 13 included studies, 8 reported rates of clinically relevant PTSD symptoms, with a pooled prevalence of 42%. Four of the 13 included studies reported rates of participants meeting PTSD diagnostic criteria; the pooled prevalence of PTSD diagnosis was 30%. Anxiety and depression were identified as potential risk factors but again the evidence for this was generally weak. The reported prevalence rates from both previous reviews vary widely (11–67%); arguments for such variation have focussed on methodological inconsistencies in assessing psychosis-related PTSD such as measurement tools used and definitions of psychosis-related PTSD [20].

### Aim

The aim of this systematic review was to review the literature on prevalence rates and associated factors of psychosis-related PTSD in people who have experienced one or more episode of psychosis. We reviewed literature from 2011 in order to update the earlier review by Berry et al. (2013) [14] and consider the extent and drivers of psychosis-related PTSD in a modern healthcare context. The current review adopted a systematic methodology similar to that used by Rodrigues and Anderson (2017) [15] but was not limited to first-episode psychosis.

Our review questions were:

1. What is the prevalence of psychosis-related PTSD in people with psychosis (who have experienced any number of episodes)?
2. What factors are associated with the development of psychosis-related PTSD?
3. Do the prevalence rates and/or associated factors differ between first-episode samples and people who have experienced multiple episodes?

## Method

We registered our protocol on PROSPERO on 24th June 2019 (registration number CRD42019138750). We followed PRISMA (Preferred Reporting Items for Systematic Reviews and Meta-Analyses) guidelines for reporting systematic reviews.

### Inclusion criteria

Studies satisfying the following criteria were included in this systematic review: (1) participants had experienced at least one episode of any type of psychosis; (2) estimated the prevalence and associates of psychosis-related trauma symptoms or PTSD diagnosis; (3) were published into journal articles. All types of study design were included. Studies that reported prevalence of PTSD not specific to psychosis were excluded, as were grey literature including doctoral theses and conference abstracts.

### Definition of key terms and concepts

We included studies with any definition of PTSD as long as it was related to the experience of psychosis (e.g. psychosis-related PTSD, post-psychosis PTSD). We included participants who met clinical diagnoses of PTSD (not restricted by diagnostic classification) and/or who scored above clinically relevant cut-offs on validated measures of PTSD. We included studies that had participants with all types of psychosis as diagnosed according to either the DSM-IV, DSM-V or ICD-10 classifications.

### Search strategy

The electronic search was conducted on the databases MEDLINE-Ovid, EMBASE-Ovid, PsycINFO-Ovid, Web of Science and the Published International Literature on Traumatic Stress (PILOTS). The search strategy for MEDLINE was adapted from previous reviews [14, 15], further refined following consultation with a medical librarian, then adapted accordingly for the other databases. Search terms were related to the concepts of ‘psychosis’ and ‘trauma’. The search was restricted to English-language journal articles published 2011 onwards, after the final search of a previous review [14]. See Additional file 1 for search strategy.

We used forward and backward citation searching and manual searching of the reference lists of key papers using Google Scholar. Where our search returned conference abstracts and doctoral theses, we contacted authors to see whether the research had subsequently been published as journal articles. A librarian was contacted to request an English translation of a non-English paper that was retrieved.

### Study selection

Two reviewers (GB and ZH) independently screened 10% of the retrieved titles and abstracts against the inclusion criteria. The reviewers discussed their chosen papers for inclusion and GB then screened the remaining titles and abstracts for further eligible papers. Both reviewers independently screened 100% of full texts that were identified as potentially eligible. GB and ZH shared their results and resolved discrepancies through discussion and consultation with reviewers JB and BLE.

### Data extraction

A data extraction tool was adapted from one used by Rodrigues and Anderson (2017) [15] and included characteristics of the studies and the main findings. GB and ZH extracted data independently to ensure accuracy and reliability of extraction. A third extraction table was created to record the related factors that each study had assessed.

### Study quality

The quality of studies was evaluated using an adapted version of the Newcastle-Ottawa Scale (NOS) [21], which was utilised in a similar review. The NOS assesses study quality based on sample selection, comparability between groups and outcome assessment [15]. GB and ZH independently developed adapted versions then agreed on the final version; items relating to follow-up were removed as they were not applicable to the included studies. Higher scores indicated greater quality. Studies were judged as low, medium or high quality if their score was between 1 and 4, 5–8, and 9–12 respectively. The highest score for representativeness could be awarded if the sample was large and included people with psychosis from different settings (e.g. site or service type) and the lowest if there was no description of setting. Studies were judged to have a higher score if their participants had a clinical diagnosis of the exposure (psychosis) and the assessment of outcome (PTSD related to psychosis) was through either a clinical interview or relevant self-report measure. Studies were judged as higher quality if they described individuals who declined participation. Whilst confounders are not relevant to the assessment of prevalence, we were also interested in factors that might be associated with psychosis-related PTSD, for which confounding does need to be considered. Therefore, studies were judged as higher quality if they adjusted for confounders. Plausible confounders were co-morbid psychopathology and PTSD not related to psychosis. The quality assessment was completed independently by GB and ZH and any discrepancies were resolved by discussion. Quality assessment was not used as a tool to exclude any studies from analysis but instead to aid a critical review of the evidence.

### Synthesis of results

We planned a priori to meta-analyse where outcomes and populations from more than three studies were sufficiently homogeneous. However, of the four studies that used the same outcome measure (the Impact of Events Scale Revised; IES-R) [30], the samples were small in size, had large confidence intervals and were from markedly different settings (patients on secure wards, compared to outpatients in early psychosis services in the other studies). Expert advice was sought from a statistician who supported our decision not to combine these studies in a meta-analysis due to heterogeneity.

A narrative approach informed by the guidance by Popay et al. (2006) [22] was used to synthesise the study findings in four stages: (1) developing a theory, (2) developing a preliminary synthesis, (3) exploring relationships within and between studies, and (4) assessing the robustness of the synthesis.

#### Stage 1: development of theory

This stage was performed early in the review process and helped to determine the current theories surrounding psychosis-related PTSD, identifying where further investigation was needed, and thus shaping the review question. Prominent theories included those of sensitisation and re-traumatisation which informed the review focus on prevalence rates in samples of people with first or multiple episodes of psychosis.

Psychological and cognitive theories described how the processing of trauma can be influenced by various individual and social factors. With psychosis-related PTSD there are two potentially different traumatic exposures: psychosis symptoms and treatment experiences. This guided our focus on associated factors as a way of exploring potential mechanisms underlying psychosis-related PTSD.

#### Stage 2: development of the preliminary synthesis

This stage involved organising and describing the included studies to be able to search for patterns across studies. Data were extracted and presented in tables.

#### Stage 3: exploring the relationships within and between studies

Stage three involved exploring relationships between studies on their key findings and methodological, clinical, and theoretical differences. To synthesise prevalence rates studies were grouped by PTSD outcome measure. The synthesis of related factors was separated into a) factors that were the primary focus of studies and the theoretical bases for these and b) secondary factors.

#### Stage 4: evaluating the robustness of the synthesis

The methodological quality of included studies and of the review process was examined to assess the strength of the evidence provided by the review. Considerations were made of the generalisability of the results to the wider population.

## Results

### Study selection

**Fig. 1:**
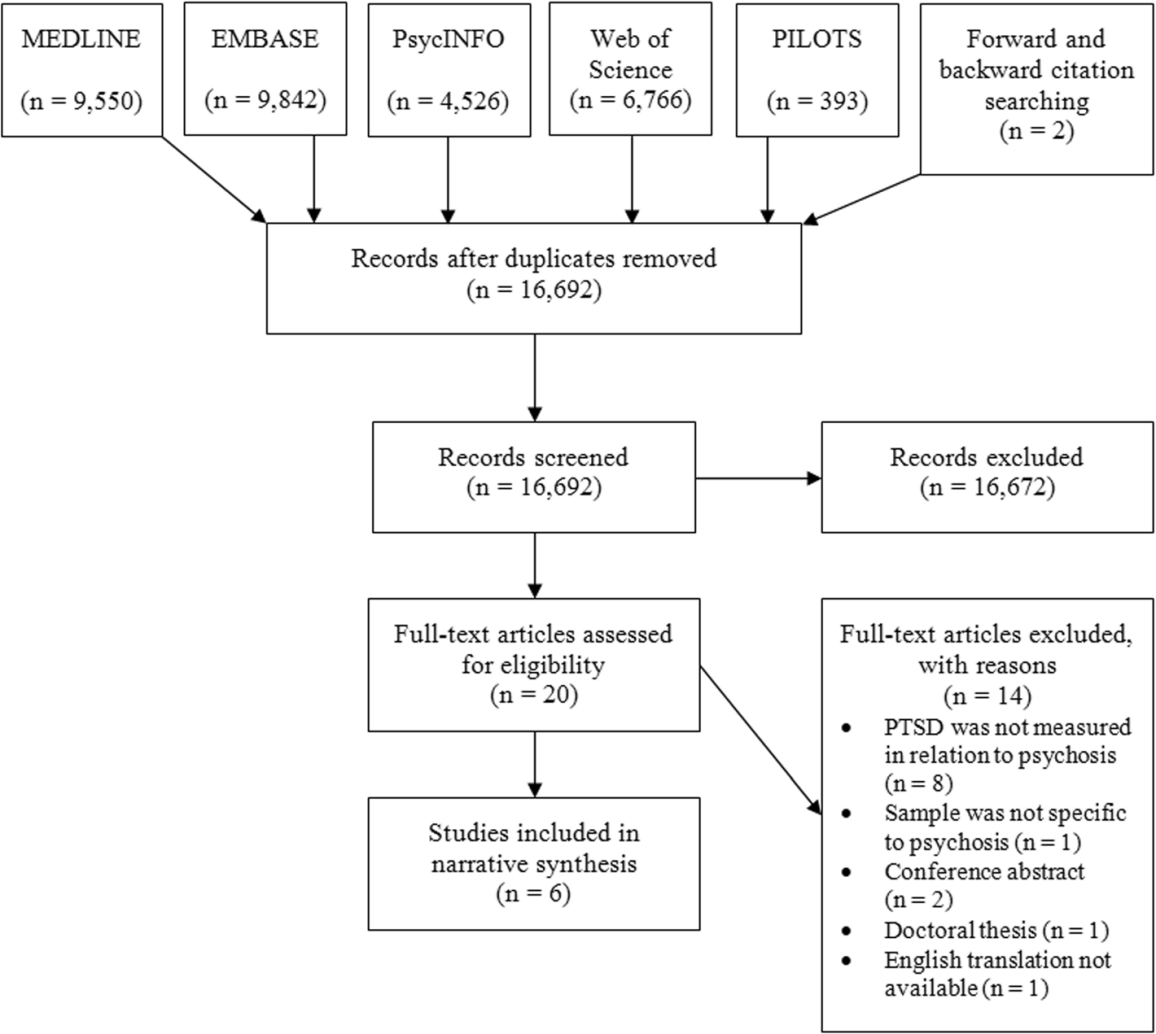
PRISMA Flowchart

Figure 1 illustrates our search strategy results (see Fig. 1). After 10% of titles and abstracts had been screened independently by reviewers, assessments of eligibility were compared and there was a disagreement between reviewers of one paper (1/5) which was discussed and then rejected. Of the 20 full-texts subsequently screened the reviewers disagreed on two (2/20). One of these was resolved by discussion and the other after consultation with a third and fourth reviewer; both were rejected. Seven papers were assessed as eligible; however, one paper was excluded during data extraction as the PTSD prevalence included cases of PTSD from non-psychosis-related events. This left six eligible papers remaining for inclusion.

### Study characteristics

We included six studies with a total of 332 participants. Sample sizes ranged from 34 [23] to 110 [24]. All studies (*n* = 6) used cross-sectional study designs. See Table 1 for characteristics of included studies (Table 1). Four studies were based in the UK [23, 24, 27, 28], one in Australia [26], and one in Tunisia [25]. Half (*n* = 3) were in early intervention in psychosis (EIP) services [23, 26, 28]. EIP services typically treat people who have experienced, or are experiencing, their first episode of psychosis. However, the length of time since onset was not reported in these studies, and the characteristics of the samples suggest they may vary considerably: in one study participants could have been recruited as soon as they were seen by early intervention services [26] and therefore could be currently experiencing their first episode, whereas in another study the participants were all in remission from their first episode [28]. A third study [23] stated that people who had experienced more than one episode could be included and therefore participants beyond first episode might have been grouped together with first episode participants.

**Table 1:** Characteristics of included studies

	Study	Study location	Study design	n	Source of sample	Mean age (SD)	Male (%)	Diagnostic criteria for psychosis	Stage of psychosis
**1**	Abdelghaffar et al. (2018) [25]	Tunisia	Cross-sectional	52	Outpatients of 1 psychiatric hospital	27.6 (5.6)	51.9%	Not specified	Within 2 years of inpatient admission for FEP
**2**	Bendall et al. (2012) [26]	Australia	Cross-sectional	36	Outpatients of 1 early psychosis service	21.42 (3.43)	61%	DSM-IV-TR	Within 18 months of first treatment for FEP
**3**	Berry et al. (2015) [27]	UK	Cross-sectional	50	Secure wards	37.66 (11.16)	80%	ICD-10 (F20, F22, F23, F25)	Inpatients 1 > month
**4**	Picken & Tarrier (2011) [24]	UK	Cross-sectional	110	Clinical trial for CBT for SUD in psychosis	38 (10)	90%	DSM-IV-TR	Not specified
**5**	Pietruch & Jobson (2012) [23]	UK	Cross-sectional	34	1 EI service	25.67 (6.04)	64.7%	Not specified	Have experienced 1+ episode in last 3 years
**6**	Turner et al. (2013) [28]	UK	Cross-sectional	50	1 EI service	24.5 (−)	Not specified	ICD-10 (F20, F22, F23, F25)	FEP in remission

*FEP* First episode of psychosis, *DSM-IV-TR* Diagnostic Statistical Manual of Mental Disorders Fourth Edition, *ICD-10* International Classification of Diseases 10th Edition, *CBT* Cognitive Behaviour Therapy, *SUD* Substance Use Disorder, *EI* Early intervention

One study was based in the outpatient department of a psychiatric hospital, where participants were within 2 years of admission for FEP but it was not clear how many episodes they had experienced or when the first episode started [25]. One was in secure wards with current inpatients [27] where it was likely the participants were mixed in terms of numbers of episodes and length since onset. One study utilised a sample of outpatients participating in a clinical trial [24], where all participants had co-morbid substance use disorder. Overall, the number of episodes experienced and the length of time since onset varied across samples and was often not made clear.

### Quality assessment

After independently scoring the adapted NOS the reviewers agreed on 29/36 (86%) of items for all studies. Disagreements were resolved by returning to the papers and further discussion. See Table 2 for results of quality assessment (see Table 2).

**Table 2:** Quality assessment of included studies based on an adapted version of the Newcastle-Ottawa scale

	Quality domain	Quality criteria	Abdelghaffar et al. (2018) [25]	Bendall et al. (2012) [26]	Berry et al. (2015) [27]	Picken & Tarrier (2011) [24]	Pietruch & Jobson (2012) [23]	Turner et al. (2013) [28]
1.	Representativeness of sample	Truly representative (2)	1	1	1	0	1	1
		Somewhat representative (1)						
		No description of derivation of sample (0)						
2.	Ascertainment of exposure	Patient notes (2)	2	2	2	2^a^	0	2
		Clinical interview (2)						
		Self-report (1)						
		No description (0)						
3.	Same method of ascertainment for entire sample	Yes (2)	2	2	2	2	0	2
		No (0)						
4.	Non-participation rate	High rate, described (2)	0	2	0	0	0	0
		Low rate, described (2)						
		All participants asked took part (2)						
		Non-participants not described (0)						
5.	Assessment of outcome	Questionnaire (2)	2	2	2	2	2	2
		Clinical interview (2)						
		Self-report or patient notes (1)						
		No description (0)						
6.	Confounders	Confounders described and adjusted for (2)	1	1	0	1	1	0
		Confounders described (1)						
		No description (0)						
	**TOTAL**		**7** Medium	**8** Medium	**6** Medium	**6** Medium	**4** Low	**6** Medium

Higher scores reflect superior quality. Scores 1–4 = low, 5–8 = medium, 9–12 = high

^a^A researcher conducted the clinical interview, not a clinician

Five out of six studies were judged as medium quality and one was low (Table 2). None were judged to be truly representative of people with psychosis due to the relatively small and restricted samples. One study did not clearly describe how participants were recruited or from what setting [24]. Two studies utilised samples that were unlikely to be generalizable to the rest of the population (patients on secure wards, 25; patients with substance use disorders, 24). The ascertainment of exposure and outcome was generally good across studies. The studies were generally poor at describing characteristics of non-participants. We expected plausible confounders to be co-morbid psychopathology and PTSD not related to psychosis, however no studies clearly described and adjusted for these; one mentioned adjusted analyses but did not report these results [28].

### Findings: prevalence

Table 3 outlines key findings of prevalence rates (see Table 3). Prevalence rates ranged from 14 to 47% for full PTSD. One study did not report an overall prevalence figure for PTSD and instead reported separate PTSD rates for individual psychosis and treatment-related experiences [24].

**Table 3:** Key findings of prevalence of psychosis-related PTSD from included studies

	Study	Trauma measure used	Criteria for psychosis-related PTSD	Treatment & symptoms separated?	Key findings of prevalence		
					Full PTSD for all causes	Full PTSD related to symptoms	Full PTSD related to treatment
1	Abdelghaffar et al. (2018) [25]	CAPS	‘Full’ PTSD: Fulfils criteria A, B, C, D	Yes + combined	42.3%	23.1%	19.2%
			‘PTSD Syndrome’: Fulfils criteria B, C, D		69.2%		
2	Bendall et al. (2012) [26]	IES-R	Exceeds clinical cut-off score^a^	No	47%	-	-
3	Berry et al. (2015) [27]	IES-R	Exceeds clinical cut-off score^a^	Yes	30%	24%	18%
4	Picken & Tarrier (2011) [24]	PDS	‘Modified-Full’ PTSD: A, B, C, D	Yes	-	14% delusions 16% hallucinations	21% involuntary admission 3% traumatic treatment
5	Pietruch & Jobson (2012) [23]	IES-R	Exceeds clinical cut-off score^a^	No	41.18%	-	-
6	Turner et al. (2013) [28]	IES-R	Exceeds clinical cut-off score^a^	No	14%	-	-
			Exceeds cut-off for partial symptoms^b^		24%		

*CAPS* Clinician-Administered PTSD Scale [29], *IES-R* Impact of Events Scale–Revised [30], *PDS* Posttraumatic Stress Diagnostic Scale [31], ^a^Cut-off score of 33 on IES-R recognised as having diagnostic accuracy for PTSD [32]. ^b^Partial symptoms: above cut-off scores for subscale of re-experiencing plus either avoidance or hyperarousal, as proposed by Asukai et al. (2002) [33]

There were variations in how the six papers conceptualised psychosis-related PTSD: studies differed in whether they separated or combined symptoms and treatments, isolated different symptoms or not, and if they reported ‘partial PTSD’ for sub-clinical levels as well as ‘full PTSD’. These differences necessitate that caution is taken in grouping together the prevalence rates across the different studies as the reported rates might not all refer to the same concept.

The measurement of psychosis-related PTSD also varied. The majority (*n* = 4) of studies used the IES-R [23, 26–28, 30]. The IES-R is a 22-item self-report measure that assesses post-traumatic stress symptoms following an event and consists of three subscales measuring the three domains of PTSD according to the DSM-IV (intrusions, avoidance and hyperarousal). Note, the DSM-V added a fourth domain (negative alterations in cognitions and mood), which is not captured by the IES-R. Item examples include “I was jumpy and easily startled” and “I tried not to think about it”. Respondents are asked to rate each item on a 5-point scale from 0 (not at all) to 4 (extremely). Higher scores indicate higher symptoms of trauma [30]. The four studies that used this measure all utilised a cut-off score of 33 to determine ‘case-ness’ of PTSD, as proposed by Creamer et al. (2003) [32], and reported the percentage of their sample that scored above the cut-off as the prevalence. Three of the four also reported the average scores for the sample; variation in scores within samples was high.

The administration of the IES-R differed across the studies. Studies requested participants answered the IES-R in relation to acute psychosis [26], the most recent episode [23] or the most traumatic experience that occurred in relation to their mental illness [28]. One asked participants to complete the measure twice: once in relation to most distressing psychotic symptom and once in relation to most distressing hospital experience [27]. The IES-R is designed as a self-report measure, but in two studies [26, 28] it was completed with the researcher. The variation in delivery of the IES-R between studies might have influenced participants to report different levels of PTSD.

One study [24] used the Post-traumatic Diagnostic Scale (PDS) [31], a self-report measure that mirrors the DSM-IV diagnostic criteria for PTSD. Participants identified their most distressing experience and then answered questions to determine whether it met criterion A for a PTSD diagnosis (i.e. it involved threat and danger and invoked negative emotional responses). The percentages of participants who met PTSD criteria for individual psychosis-related events were: delusions (14%), hallucinations (16%), traumatic treatment (3%) and involuntary admission (21%). These individual percentages could not be compared to the total PTSD prevalence rates in the other studies.

The remaining study [25] employed the Clinician-Administered PTSD Scale (CAPS; [29]) to ascertain prevalence in their sample. The CAPS is a semi-structured clinician-administered interview designed to assess PTSD as defined by the DSM-IV. Participants were asked to consider the experience of psychosis symptoms and of treatment separately; the prevalence rates were 23.1 and 19.2% respectively, so 42.3% of their sample met full PTSD criteria for a psychosis-related event. No participants met full PTSD criteria for both symptoms and treatment. The overall prevalence rate is comparable to those reported by studies using the IES-R with the exception of the considerably lower rate reported by Turner et al. (2013) [28].

### Findings: associated factors

Four studies hypothesised that psychosis-related PTSD would be associated with a key factor, based on an underlying theory. All studies also tested for associations between secondary factors and psychosis-related PTSD. See Table 4 for a summary of all factors.

**Table 4:** Summarised results from included studies of factors potentially associated with psychosis-related PTSD

Potentially associated factors	# of studies tested associations	# of significant associations
*Demographics*		
Age	1	0
Gender	2	0
*Psychosis characteristics*		
Diagnosis	1	0
Age of onset	1	0
Years since onset	1	0
DUP	2	0
Time since last episode	1	0
Psychosis severity	4	2
Positive symptoms^a^	2	2
Negative symptoms^a^	1	0
General psychopathology^a^	2	0
*Treatment experiences*		
Restraint	1	1
Threat by other patients	1	0
Threat by treatment provider	1	0
Involuntary hospitalisation	1	0
Medication side effects	1	0
Length of current admission	1	1
No. of hospitalisations	1	0
No. of traumatic hospital events	1	1
*Previous trauma experience*		
Lifetime trauma	2	0
Childhood trauma	1	1
*Other clinical factors*		
Depression	3	3
Global functioning	1	0
Substance use	2	0
PTSD related to childhood trauma	1	1
*Coping styles*		
Maladaptive coping	1	1
*Disclosure of trauma*		
Urge to talk	1	0
Reluctance to talk	1	1
Actual self-disclosure	1	1
*Experiences of shame*		
Internal shame related to psychosis	1	1
External shame related to psychosis	1	1
General shame	1	1
*Attachment*		
Anxiety	1	0
Avoidance	1	1

*DUP* Duration of Untreated Psychosis. ^a^Subscale of the PANSS

### Key factors

The key factors examined were childhood trauma [26], attachment style [30], disclosure of trauma [23] and feelings of shame [28]. Bendall et al. (2012) [26] hypothesised that childhood trauma was a moderator between psychosis and psychosis-related PTSD. This was based on the theory that early traumas can sensitize individuals to develop PTSD after a later trauma, in this case psychosis. Their results supported both hypothesis and theory as they found that experiencing childhood trauma increased the risk of psychosis-related PTSD by 27 times (*p* = 0.01, 95% CI: 2.96–253.80). Childhood trauma-related PTSD also increased risk (OR 20.40; 95% CI 3.38–123.25, *p* = 0.01; *r*2 = 0.45).

Berry et al. (2015) [27] focussed on attachment theory and hypothesised that insecure attachments (which affect mental representations of the self and others [34];) might be influential in developing PTSD from psychosis. They found that anxious attachments were positively correlated with both psychosis-related (*B* = 0.40, 95% CI: 0.54–2.28) and hospital-related (*B* = 0.41, 95% CI: 0.51–2.23) PTSD symptoms. Pietruch and Jobson (2012) [23] theorised that self-disclosure of trauma is important in recovery and posttraumatic growth. Their hypothesis was supported; reluctance to talk and actual self-disclosure were positively and negatively associated with psychosis-related PTSD, respectively (*r* = .42, *p* = .02; *r* = −.43, *p* = .01).

Turner et al. (2013) [28] focussed on theories which proposed that experiencing shame can be socially and psychologically damaging. They suggested that individuals with psychosis may experience shame through having a highly stigmatised illness, and that shame might explain PTSD following interpersonal traumas. Their results indicated a correlation between psychosis-related PTSD and both internal (*r* = .48, *p* < 0.01) and external (*r* = .64, *p* < 0.01) shame associated with psychosis, as well as general shame (*r* = .57, *p* < .001).

Overall each of the four papers received some evidence to support their hypotheses and consequently the underlying theories. The theories are all psychological or social in nature, and findings suggest how psychosis-related PTSD might arise either due to the effect of early life experiences on the development of the mind or the way an individual relates to others.

### Secondary factors

Depression was the only factor found to be associated in all studies that assessed it [24, 25, 28]. Secondary factors that were found to be associated in at least one study were symptom-related (severity of psychosis, positive symptoms, and general psychopathology), treatment-related (restraint, length of admission, number of traumatic hospital events), depression and maladaptive coping.

There was inconsistent evidence between studies on psychosis severity and trauma history. Psychosis severity was measured by the Positive and Negative Syndrome Scale (PANSS) [35] in four studies. Berry et al. (2015) [27] and Picken and Tarrier (2011) [24] found that the PANSS total score and subtotals for positive symptoms and general psychopathology were all associated with psychosis-related PTSD. Abdelghaffar et al. (2018) [25] and Bendall et al. (2012) [26] both only looked at the PANSS total score and reported no association.

Childhood trauma was associated in one study [26] but lifetime trauma was not in two studies [25, 27]. In the study by Abdelghaffar et al. (2018) [25], only 40% of the sample reported experiencing a traumatic event so there might not have been enough data to detect an association in this sample. However, in the study by Berry et al. (2015) [27], 94% reported at least one traumatic event yet this was also not associated with psychosis-related PTSD. It could be that adulthood trauma is not associated but childhood trauma is; this could be explained through the effect of trauma on the developing child, such as attachment style.

## Discussion

The findings of this systematic review suggest that between 14 and 47% of people with psychosis might experience psychosis-related PTSD. Depression was most commonly associated with psychosis-related PTSD. Other factors that were associated in at least one study were: symptom-related (severity of psychosis, positive symptoms, and general psychopathology); treatment-related (restraint, length of admission, number of traumatic hospital events); childhood trauma and childhood trauma-related PTSD; reactions to the trauma (maladaptive coping, reluctance to talk, actual self-disclosure); and other individual-level factors (experiences of shame, anxious attachment).

The number of studies included in this review (six) was considerably smaller than in previous reviews, which included 24 [14] and 13 [15] studies, many of which were published before 2011. We did not find, as we had expected, that more studies had been published since 2011 in line with the increasing interest in trauma in mental health research. We also found few studies clearly looking beyond the first episode; most of the studies were set in early psychosis services and/or did not describe their sample with sufficient detail. Due to this, we were unable to examine psychosis-related PTSD across the course of psychosis as we had planned.

The prevalence rates we found were similar to the rates of 11–67% reported by Berry et al. (2013) [14] and the pooled prevalence estimates of 30% (PTSD diagnosis) and 42% (PTSD symptoms) reported by Rodrigues and Anderson (2017) [15]. Our review and both previous reviews found wide variations in reported prevalence rates. In our narrative synthesis we examined differences in the conceptualisation, definition and assessment of psychosis-related PTSD between the included studies and it is likely that these factors can provide some explanation for variations in prevalence rates across all three reviews [20]. Other factors such as differences in participant populations, in the amount of cumulative exposure to traumatic psychosis and in service provision experienced, may also account for the wide variations in prevalence rates between studies across all three reviews. Similar to our findings, both previous reviews listed trauma history, psychosis severity and depression as possible related factors. They also noted that sample sizes were possibly too small to detect associations and reliably estimate prevalence.

The findings of our review largely corroborate the findings in the previous review by Berry et al. (2013) [14]. However, the Berry et al. (2013) [14] review was limited in that it was not systematic. As our review used systematic methodology it provides more robust evidence for prevalence rates and factors associated with psychosis-related PTSD. Additionally due to the rigorous systematic methods we employed, we can say with reasonable certainty that the low number of papers retrieved reflects the lack of recent research, rather than the possibility that papers were missed. Importantly, our review has highlighted that there have been few studies conducted on this topic over the past decade, despite appeals that further research is required. For example, the authors of the earlier review stated that future research should use sensitive measures of trauma, separate out symptom and treatment-related PTSD, and that psychological processes should be investigated. The present review has found that largely, these recommendations have not yet been met. Some studies have separated out symptom and treatment-related PTSD, but this is not consistent. Some of the papers elicited in our review focussed on psychological processes which were hypothesised to be important in the development of psychosis-related PTSD, but most did not.

The review by Rodrigues and Anderson (2017) [15] was systematic and used meta-analysis; however, they too were limited by a small number of studies to analyse (the subgroups included in meta-analyses were made up of 8 and 4 studies). This previous review was solely focussed on first episode psychosis so it has not hitherto been known whether the first episode of psychosis is more or less traumatic than subsequent episodes. The present review had a broader scope by including studies across the course of psychosis and as a result we retrieved studies not included in the review by Rodrigues and Anderson (2017) [15]. Those additional studies provided data that allowed us to look closely at theories underpinning the development of psychosis-related PTSD (e.g. attachment theory; [27]) and suggested how separate experiences may lead to PTSD symptoms (e.g. by separating delusions and hallucinations; [24]).

### Strengths and limitations of included studies

Due to the cross-sectional designs, we cannot infer causation of associated factors. Prospective research is required and is possible. One of our excluded studies recruited patients during the acute stage of psychosis and then followed them up 18 months later, allowing them to investigate whether psychosis-related factors were predictors of PTSD [36]. Their measurement of PTSD was not specific to psychosis-related events so this study had to be excluded, however its prospective methodology is noteworthy. Many included studies did not adjust for plausible confounders, such as non-psychosis-related PTSD. Most of the sample sizes were small and limited to one service. Studies which reported statistically significant associations [26] had very large confidence intervals indicating high variance within the samples. With sample sizes this small it is difficult to generalise the findings.

A limitation in this field is a lack of agreement whether trauma related to symptoms and trauma related to treatment are both ‘psychosis-related’ and whether distinctions between these should be made when collecting data. Differences between studies on how the same measurement tool was used might have elicited different rates of PTSD, and this variability between studies on the concept of psychosis-related PTSD presents complications in comparing prevalence rates and associated factors between different studies.

The measurement tools used were generally psychometrically robust and validated, and the questionnaires had been reliably used with psychosis populations. The use of a clinician-administered scale in one study, the CAPS, is positive as this is considered the gold-standard for measuring PTSD. However, interrater reliability was not assessed, and the CAPS was translated into Tunisian-Arabic for this study but was not validated in that cultural context. Most of the studies included did not sufficiently describe their non-participation rate; individuals who chose not to participate in research about trauma might have declined precisely because they have PTSD, therefore there is a risk of sampling bias across the studies.

Potentially relevant factors were not investigated for associations with psychosis-related PTSD. Firstly, ethnicity: research suggests that people from black and minority ethnic (BME) backgrounds are considerably more likely to be diagnosed with psychosis [37] and to receive coercive treatment [38] than other ethnicities. They could therefore be particularly vulnerable to traumatic psychosis-related experiences. However, none of the included studies assessed for associations between ethnicity and psychosis-related PTSD.

Treatment-related factors were somewhat neglected across the studies and only one study assessed correlations with involuntary hospitalisation and restraint [25]. Coercive practices are potentially modifiable but the paucity of research into treatment factors limits understanding of their traumatic nature and potentially reductions in their use.

Some known risk factors for PTSD were not assessed in the included studies. Predictors of PTSD are reported to include perceived threat, intense emotions and dissociation during the traumatic event, and low perceived social support after the event [12, 13]. Perceived threat was partly investigated by Abdelghaffar et al. (2018) [25] who assessed perception of threat from other patients and care providers. Pietruch and Jobson (2012) [23] investigated disclosure of trauma, which is one aspect of social support; however, social support might protect against PTSD in more ways than encouraging people to talk about their trauma. Intense emotions and dissociation during psychosis were not assessed in the included studies.

### Strengths and limitations of the review

Our review was restricted to papers published from 2011 onwards which resulted in only a small number of studies being retrieved. However, this allowed us to provide an updated evidence review and to look more closely at the extent and drivers of psychosis-related PTSD in a modern healthcare context. Our inclusion criteria resulted in the exclusion of a doctoral thesis due to non-peer review, and a paper written in French as we were unable to translate it. Both of these may have contributed to the findings in this review had they been included. However, our search strategy was broad, so it is unlikely we missed relevant papers; we searched five databases, used over-inclusive search terms, and a second reviewer assisted with the screening of the search output.

The small number of included studies prevented the ability to carry out analyses of subgroups as planned a priori in the PROSPERO protocol. However, finding only six additional studies than the previous reviews [14, 15] reflects the lack of research published in the field since 2011 despite recommendations for further research, rather than being a limitation of this review per se. This review has highlighted that further studies of prevalence and associated factors are required, with distinctions made between FEP and multiple-episode psychosis, and that greater clarity and consistency in how psychosis-related PTSD should be defined and assessed is necessary to reliably combine results from multiple studies.

We adapted a quality assessment tool because we could not find a more appropriate, validated tool for this review. This could have been further adapted to include some factors specifically relevant to the assessment of PTSD, such as whether sufficient amount of time had lapsed since traumatic event for a diagnosis of PTSD [20]. However, the tool we used was utilised in a similar adapted form in previous studies [15]. The adaptation of the tool to fit our criteria was assisted by a second reviewer independently, reducing risk of bias. The adaptation resulted in the removal of all follow-up criteria due to irrelevance to the research question.

We included a study [24] which did not report an overall prevalence rate for psychosis-related PTSD, but instead separate rates for different psychosis-related events (e.g. delusions, hallucinations, involuntary hospitalisation). These figures could not be directly compared with prevalence rates from other studies. However, we decided to include this study as it does provide relevant data on people meeting PTSD criteria based on their psychosis experience.

This review built upon previous reviews [14, 15] by exploring the underlying theories of some of the associations between psychosis and related PTSD (e.g. attachment theory, trauma sensitization theory, theories of shame and disclosure of trauma). This can hopefully support the future development of a model of the processes by which psychosis-related PTSD might occur.

### Implications in research, theory and practice

Studies with prospective designs and larger sample sizes from a wider variety of settings are needed. Research should distinguish between people who have had one or multiple episodes, to investigate a potential cumulative effect of trauma from psychosis, and assess more potential risk factors, such as various treatment factors, social support, dissociation, intense emotions and ethnicity.

The wide variation in reported prevalence rates for psychosis-related PTSD is hard to interpret. Moreover, the rates of psychosis-related PTSD reported in some studies in our review are higher than rates of PTSD from any cause among people with psychosis reported in other recent studies, which did not distinguish rates of psychosis-related PTSD and were therefore not included in our review [39, 40]. We need more studies in a variety of settings and clinical populations, and more consensuses on gold-standard PTSD measures, to be able to understand how much in the variance of psychosis-related PTSD may be an artefact of inconsistent measurement approaches, and how much reflects genuine variation in clinical morbidity.

Some existing psychosocial theories might explain mechanisms underlying psychosis-related PTSD and could in the future form part of an integrated model of psychosis-related PTSD; however before this is possible there needs to be exploration of societal, environmental, cultural, and neurobiological factors.

Currently, an episode of psychosis does not fulfil criterion A in the DSM-V for a traumatic event which requires exposure to actual or threatened death, serious injury, or sexual violence [41]. It has been argued that this criterion should be expanded to include internally experienced events such as psychosis as it is the perception of threat that is necessary for PTSD [42]. Proposals for the ICD-11 will allow flexibility in the judgement of either an objective or subjective traumatic event [43]. In our review, the rates of people meeting PTSD symptom criteria following the experience of psychosis provides further support for the inclusion of subjective threat as a qualifying traumatic event that satisfies criterion A in the DSM-V classification.

Rates of psychosis-related PTSD do not appear to have reduced since 2011. The ongoing development of TIC has the potential to reduce traumatic experiences associated with psychosis, such as the use of coercive practices [16]. However, coercive practices appear to be increasing in the UK [44], particularly for people with psychosis [45]. In addition to reduced distressing treatment practices, TIC involves services recognizing that the experience of psychosis can be traumatic, screening patients for PTSD, and offering evidence-based treatments (which, NICE guidelines stipulate should commence promptly, [46]). Well implemented TIC may not directly lead to a reduction in reported rates of psychosis-related PTSD in practice, as it may conversely lead to an increase in case identification, but it would be expected that rates would decline in cross-sectional studies. It is currently not clear to what extent TIC is being delivered in clinical services. It remains crucial that TIC be developed and implemented and that services recognise the traumatic experience of psychosis and achieve early identification of psychosis-related PTSD followed by effective treatment.

## Conclusions

We must be cautious in drawing conclusions from this review as there were only a small number of studies with methodological issues. However, this review has indicated that psychosis can be traumatic enough to lead to PTSD in some individuals, and there are some factors which are associated with psychosis-related PTSD, such as depression. Further research is certainly needed, but awareness needs to be raised amongst clinical settings of the potentially traumatising experience of psychosis so that these can be addressed in treatment and through modifying care practices. Routine enquiry about childhood trauma as part of a TIC approach could also help to identify those that may be at higher risk of developing PTSD in psychosis. Efforts must be made across clinical and research settings to ensure that TIC is being delivered and to examine its effectiveness at reducing or preventing rates of trauma.

## Supplementary Information

**Additional file 1.** Medline search strategy.

## Data Availability

Data sharing is not applicable to this article as no datasets were generated or analysed during the current study.

## Abbreviations

PTSD: Post Traumatic Stress Disorder
PROSPERO: International Prospective Register of Systematic Reviews
PRISMA: Preferred Reporting Items for Systematic Reviews and Meta-Analyses
DSM-V: Diagnostic and Statistical Manual of Mental Disorders - Fifth Edition
DSM-IV: Diagnostic and Statistical Manual of Mental Disorders - Forth Edition
ICD-10: International Classification of Diseases – 10th Edition
PILOTS: Published International Literature on Traumatic Stress
NOS: Newcastle-Ottawa Scale
EIP: Early Intervention in Psychosis
FEP: First Episode of Psychosis
IES-R: Impact of Events Scale – Revised
PDS: Post-traumatic Diagnostic Scale
CAPS: Clinician-Administered PTSD Scale
PANSS: Positive and Negative Syndrome Scale
BME: Black and Minority Ethnic
TIC: Trauma Informed Care
