# Supplementary material for: A systematic review of PTSD to the experience of psychosis: prevalence and associated factors
Source: BMC Psychiatry. 2021 Jan 7;21:9. doi: 10.1186/s12888-020-02999-x (PMC7789184; doi:10.1186/s12888-020-02999-x)
Supplement: Supplementary file 1 — Additional file 1. Medline search strategy. [file 12888_2020_2999_MOESM1_ESM.docx]

**Additional File 1: Medline search strategy***

**Exposure**

Exp Psychotic disorders/

Exp Schizophrenia/

Affective disorders, psychotic/ or paranoid disorders/

Schizophrenia, Childhood/

Hallucinations/

Delusions/

(psychos$ or psychotic). ti,ab.

Schizophreni$. ti,ab.

(sever$ mental ill$ or sever$ mental disorder$ or SMI$).ti,ab

**Outcome**

Exp Stress, Psychological

Psychological trauma/ or stress disorders, post-traumatic/ or stress disorders, traumatic, acute/

Stress disorders, Traumatic/

Trauma$.ti,ab

PTSD.ti,ab

Posttraumatic stress.ti,ab.

Limit yr 2011-Current

Limit to English language
